# Congenital adhesion band causing small bowel obstruction: What’s the difference in various age groups, pediatric and adult patients?

**DOI:** 10.1186/s12893-016-0196-4

**Published:** 2016-12-07

**Authors:** Kwang-Ho Yang, Tae-Beom Lee, Si-Hak Lee, Soo-Hong Kim, Yong-Hoon Cho, Hae-Young Kim

**Affiliations:** 1 Department of Surgery, Pusan National University Yangsan Hospital, Yangsan, Korea; 2 Research Institute for Convergence of Biomedical Science and Technology, Pusan National University Yangsan Hospital, Yangsan, Korea; 3 Department of Surgery, Pusan National University School of Medicine, Geumo-ro 20, Mulgeum-eup, 50612 Yangsan, Gyeongnam Korea

**Keywords:** Congenital adhesion band, Obstruction, Pediatric, Adult

## Abstract

**Background:**

A congenital adhesion band is a rare condition, but may induce a small bowel obstruction (SBO) at any age. However, only a few sporadic case reports exit. We aimed to identify the clinical characteristics of congenital adhesion band manifesting a SBO stratified by age group between pediatric and adult patients.

**Methods:**

The medical records of all patients with a SBO between Jan 1, 2009 and Dec 31, 2015 were retrospectively reviewed. Cases associated with previous surgical procedure and cases of secondary obstruction due to inflammatory processes or tumor and other systemic diseases were excluded. The patients were divided into two groups according to age below or above 18 years: pediatric and adult. The basic clinical characteristics were analyzed and compared between groups.

**Results:**

Of 251 patients with a SBO, 15 (5.9%) met the inclusion criteria; 10 cases in pediatric group (mean age 17.9 ± 38.7 months) and 5 cases in adult group (mean age 60.0 ± 19.7 years). The pediatric group (66.6%) included 3 neonates, 5 infants, and 2 school children. They usually presented with bilious vomiting (50.0%) and abdominal distention (60.0%), and demonstrated a high rate of early operation (80.0%) and bowel resection (70.0%). In contrast, the adult group (33.3%) presented with abdominal pain (100%) in all cases and underwent a relatively simple procedure of band release using a laparoscopic approach (60%). However, group differences did not reach statistical significance. In addition, two groups did not differ in the time interval to the operation or in the range of the operation (*p* = 0.089 vs. *p* = 0.329). No significant correlation was found between the time interval to the operation and the necessity of bowel resection (*p* = 0.136). There was no mortality in either group.

**Conclusions:**

Congenital adhesion band is a very rare condition with diverse clinical presentations across ages. Unlike adult patients, pediatric patients showed a high proportion of early operation and bowel resection. A good result can be expected with an early diagnosis and prompt management regardless of age.

## Background

Small bowel obstruction (SBO) remains a common problem in the field of abdominal surgery. SBO may arise from various causes including extrinsic (adhesion, hernia, metastatic tumor, inflammatory processes, aneurysm, and unusual endometriosis) or intrinsic processes (bowel wall tumor, Crohn’s disease, intussusception, bezoar, gallstone, and foreign body) [1]. Postoperative adhesions are the most common cause, accounting for nearly 80% of all clinical cases, even with the advent of minimal invasive surgery [2, 3].

SBOs not related to the above-mentioned conditions are rarely encountered and one of the rarest causes of intestinal obstruction is a congenital adhesion band, previously referred to as an anomalous congenital band. A congenital adhesion band is an intraperitoneal adhesion that has no relation to an intra-abdominal process (previous laparotomy, inflammatory diseases, peritonitis, embryogenic remnants, etc.) and is considered to have a congenital or de novo origin. Congenital adhesion bands may cause a SBO by trapping an intestinal loop between the band and the mesentery [4].

In general, the diagnosis of a SBO is dependent on a focused history and basic physical examination. Although imaging and laboratory studies are important adjuncts, imaging studies are less useful for the diagnosis of a SBO induced by a congenital adhesion band, and could result in the delay of definite management for the SBO affecting the prognosis. Thus, operations are more likely to be needed for the diagnosis and treatment SBOs caused by a congenital adhesion band.

Previous studies of congenital adhesion bands causing SBOs consist of sporadic case reports of pediatric or adult patients [5–8]. Therefore, the present study was conducted to identify the clinical characteristics associated with congenital adhesion band manifesting a SBO in different age groups (adult and pediatric). Furthermore, the clinical implications of the age-related findings are discussed.

## Methods

### Subject selection

A retrospective review of 251 patients managed for a SBO at our institution between Jan 2009 and Dec 2015 was performed. Patients with a medical history of previous surgery were excluded. In addition, cases which occurred during an immediate postoperative course, cases of secondary obstruction due to inflammatory processes or tumor, and other systemic diseases were excluded as well. The final sample consisted of 15 cases of SBO that met criteria for the diagnosis of a congenital adhesion band. This study was approved by the Institutional Review Board (IRB No. 05-2016-110) and the data have been managed with personal information protection.

### Data extraction and analysis

Clinical characteristics including demographics, clinical presentations, preoperative radiologic studies, the time interval to the operation, operative finding and surgical procedure, and postoperative results were examined. Classified patients by age below or above 18 years, the pediatric and adult groups, then two groups were evaluated for differences in clinical factors, including the time interval to the operation and surgical procedure. In addition the association between the time interval to the operation, early vs. delayed operation (cases with conservative management for more than 2 days) and the extent of surgical procedure were examined.

Statistical analyses were performed using IBM SPSS Statistics v23 (IBM SPSS Statistics, Feltham, UK). In addition, a Fisher’s exact test was performed to evaluate the association between the time interval to the operation and the extent of the operation. A *p*-value <0.05 was considered significant.

## Results

During the study period, the incidence of a SBO due to a congenital adhesion band was 5.9% (15/251). Ten patients (66.7%) were in the pediatric group with a mean age 17.9 ± 38.7 months (3 neonates, 5 infants, and 2 children) and 5 patients (33.3%) were in the adult group with a mean age 60 ± 19.7 years. There was no sex predominance; the sex ratio was 3:2 (male to female) in both groups (Table 1).

**Table 1:** Demographic findings

Age group	N (%)	Sex (M/F)
Pediatric patients (mean age 17.9 ± 38.7 months)		
Neonate (<1 month)	3 (20.0)	6/4
Infant (1 month – 2 years)	5 (33.3)	
Childhood (2 – 12 years)	2 (13.4)	
Adolescent (12 – 18 years)	-	
Adult patients (mean age 60 ± 19.7 years)	5 (33.3)	3/2

### Pediatric cases

Bilious vomiting and abdominal distention were common symptom in the pediatric group, presenting in more than half of the patients (Table 2). A radiologic assessment other than simple radiography was performed before the operation for some pediatric cases; computed tomography (CT) was performed in 5 cases, ultrasonography (US) in 1 case, and a contrast barium study for colon in 2 cases. The time interval to the operation after symptomatic presentation was varied according to the clinical situations, with half of the patients receiving a prompt surgical management. The ileum was the most common location of the obstruction (7/10, 70.0%) and the obstruction was caused by a fibrotic band formed between the surrounding mesentery. Moreover, there were complicated cases requiring a resection, including cases with a volvulus or strangulation (4/10, 40.0%), and segmental resection was performed in 7 cases (70.0%) (Table 3). There were no postoperative complications.

**Table 2:** Clinical presentations

Symptom	Pediatric group (*n* = 10), N (%)	Adult group (*n* = 5), N (%)
Vomiting		
Non-bilious	3 (30.0)	1 (10.0)
Bilious	5 (50.0)	-
Abdominal distention	6 (60.0)	2 (20.0)
Abdominal pain	2 (20.0)	5 (100.0)
Hematochezia	1 (10.0)	-

**Table 3:** Pediatric group: clinical findings, surgical procedure and complication

Case (Sex/Age group at operation)	Other radiologic study	Interval to operation	Operative findings	Obstruction level	Procedure
1 (M/I)	CT	1 day	thin fibrotic band between ileal mesentery and cecum	ileum	SR
2 (F/I)	CT	prompt surgery	thick band at mesenteric base extending to right upper abdominal wall multiple thin interloop bands	jejunum	BR
3 (M/N)	US	prompt surgery	band from mesenteric root compressing ileum	volvulus of terminal ileum	SR
4 (F/I)	Contrast barium enema	2 months	fibrotic band at terminal ileum	ileum	SR
5 (F/I)	CT	prompt surgery	fibrotic band at terminal ileum	volvulus of terminal ileum	SR
6 (M/N)	Contrast barium enema	1 days	fibrotic band between mesenteric root and distal ileum	ileum	SR
7 (M/N)	-	prompt surgery	multiple thin interloop bands	volvulus of jejunum	SR
8 (M/I)	-	1 month	fibrotic band at terminal ileum	ileum	BR
9 (M/C)	CT	1 day	fibrotic band between mesenteric root and jejunum	jejunum	BR
10 (F/C)	CT	prompt surgery	internal hernia due to fibrotic band between mesenteric root and distal ileum	Strangulation of ileum	SR

*I* infant, *N* neonate, *C* childhood, *CT* computed tomography, *US* ultrasonography, *SR* segmental resection, *BR* band release

### Adult cases

All of the adult patients presented with abdominal pain (Table 2). In the adult group, a surgical management was determined after conservative care for several days. For all patients, a CT scan was performed. The obstruction was located in the ileum for all patients. The fibrotic band arose from a sigmoid colonic wall in 2 cases. Surgical management was relatively simple; only a band release with a laparoscopic procedure was performed in 3 cases (60.0%) and a segmental resection was performed in 2 cases (40.0%). Generally, a simple fibrotic band had formed around the obstruction site (Table 4). There were no postoperative complications.

**Table 4:** Adult group: clinical findings, surgical procedure and complication

Case (Sex/Age range at operation)	Other radiologic study	Interval to operation	Operative findings	Obstruction level	Procedure
1 (F/71 – 80 years)	CT	4 day	fibrotic band between mesenteric base and sigmoid colon	ileum	BR
2 (F/71 – 80 years)	CT	2 days	fibrotic band between ileal mesentery and terminal ileum	terminal ileum	SR
3 (M/21 – 30 years)	CT	4 days	fibrotic band between ileal mesentery and terminal ileum	ileum	L-BR
4 (M/51 – 60 years)	CT	2 days	thick fibrotic band between terminal ileum and sigmoid colon	ileum	L-BR
5 (M/61 – 70 years)	CT	1 day	fibrotic band between ileal mesentery and distal ileum	ileum	SR

*CT* computed tomography, *BR* band release, *SR* segmental resection, *L-BR* laparoscopic band release

### Comparison between groups (Table 5)

**Table 5:** Comparison of the time interval to the operation, the range of operation, and the correlation of the time interval to the operation with bowel resection according to age group

Comparison variables	Pediatric group, N	Adult group, N	*p*- value
Time interval to the operation			
Early	8	1	0.089
Delay	2	4	
Extent of surgical procedure			
BR	3	3	0.329
SR	7	2	
Relation with bowel resection			
Early/SR	6	1	
Early/BR	2	-	0.136
Delay/SR	1	1	
Delay/BR	1	3	

Early, cases performing operation at no more than one day; Delay, cases with a conservative management for more than 2 days

*BR* band release, *SR* segmental resection

The pediatric group tended to undergo surgery at an earlier stage (clinical situations indicated conservative management for no more than one day) and were more likely to undergo segmental resection compared to the adult group; however, the differences were not significant (*p* = 0.089, 0.329 respectively). In addition, there was no significant correlation between the time interval to the operation and the necessity of bowel resection across all patients (*p* = 0.136).

## Discussions

Although congenital adhesion bands are usually identified in pediatric patients, they may give rise to a SBO at any age. The incidence rate for congenital adhesion bands is still uncertain. The incidence of adhesion without previous operations has been reported to range from 3.3 to 28% as determined by autopsy [9, 10]. Although the present study is limited by regional restrictions, an incidence of 5.9% (15/251) was found. The clinical manifestations of a congenital adhesion band vary from a mild symptomatic presentation to strangulation of the bowel, which requires a prompt surgical procedure. However, a definite preoperative diagnosis is difficult as there are no specific tests to diagnose a congenital adhesion band. Excluding other factors that may cause intestinal obstruction is currently the best diagnostic method. For these reasons, delayed diagnosis and treatment frequently occur in patients with an intestinal obstruction due to a congenital adhesion band. CT has been used to exclude other diseases in many cases [11–13], as well as in the present study. Ultimately, exploration is mandatory for both diagnosis and treatment. Moreover, the diagnosis depends on a high index of suspicious mechanical obstruction especially for patients without a history of previous abdominal surgery.

The present study revealed several interesting clinical features that varied according to age. With younger patients, there was a greater tendency for cases to be complicated with a volvulus or strangulation, and surgical management was performed in the early stage. Moreover, the extent of the surgical procedure was wider in the pediatric group compared to that in adult group. However, no statistically significant differences between the two groups were found. This may have been due to the small number of cases in each age group given the rarity of the clinical occurrence.

Previous studies have reported that the most common anatomical location of a congenital adhesion band is around the terminal ileum, followed by the mesentery root, jejunum, liver, and omentum [5–8]. Consistent with these previous studies, the present study also found that in both age groups, the band was most commonly located around the ileal mesentery and mesentery root. However the location of band does not appear to affect the degree of clinical presentation or the management.

The origin of congenital adhesion bands has an embryologic basis, such as the persistent or incomplete regression of the fetal vitelline circulation or a remnant of the ventral mesentery theory, and may be associated with genetic defects that impair embryogenesis [14–16]. In addition, other factors may be related to the formation of the band, such as an intrauterine mesothelioma trauma [17]. Congenital adhesion bands might be also a result of intrauterine exposure to certain infectious agents or ischemic events. Several reports have demonstrated evidence for an immunological mechanism in both in-vitro and in-vivo experiment [17–19]. Considering the embryologic origin, we could assume that a congenital adhesion band exists from birth and so may induce a clinical presentation earlier. Although these factors could explain the pediatric cases, they do not appropriately explain the adult cases. Instead, the adult cases could be explained as a de novo adhesions, which are shown in an autopsy study [9, 20]. Thus, the difference in clinical features between the age groups seen in the present study may be related to the multifactorial processes underlying the development of a congenital adhesion band.

Additionally, congenital adhesion bands may cause an obstruction by an internal hernia, which has usually been reported in sporadic pediatric cases [11, 21]. However, in the present study, clinical cases ranged from the neonates to the elderly, and only one case of internal hernia was in the pediatric group. The case with an internal hernia showed a severe clinical situation with strangulation of the involved segment, which required a prompt surgical resection. The present study was slightly different from previous studies with respect to management, especially in the pediatric group, as there was a tendency to perform an early operation and segmental resection [4, 6]. This may have resulted from the relatively high proportion of neonates and infants in the pediatric group, because SBOs in these age groups are associated with a high failure rate for conservative management and typically proceed to surgical management [22–24]. Furthermore, previous studies have reported that the surgical management, which includes a bowel resection, is higher for those with a younger age and a longer time interval to the operation more than 2 days [25]. However, the present study revealed no significant correlation between the time interval to the operation and the necessity of bowel resection.

Recently, a laparoscopic procedure has been increasingly used in cases of SBO with a high success rate (46 ~ 87%) [26–31]. We have also tried a laparoscopic approach in a few adult cases with good results, but have not tried this approach in pediatric case due to a limited working space and a high risk of bowel injury in pediatric patients. However, a laparoscopic procedure could be an excellent method for the diagnosis and subsequent management in cases of a SBO caused by a congenital adhesion band. Given the difficulties in diagnosis, it is necessary to attempt a laparoscopic procedure aggressively in selective cases regardless of age. Mortality associated with a SBO has been reported to be less than 10% [32, 33]. A high mortality rate is mainly related to a delay in diagnosis, which has decreased over the years, and is also associated with cases accompanying a severe underlying disease.

The present study has some limitations, mainly concerning the number of cases, which came from a single center experience with regional restrictions. However, considering that congenital adhesion bands are an uncommon cause of SBOs, the reported findings for the different age groups provide valuable clinical information.

## Conclusions

A congenital adhesion band comprises a broad spectrum of disease with different etiologies. Although it is a very rare condition with diverse clinical presentations across age groups, a good result can be expected with an early diagnosis and prompt management. Therefore, congenital adhesion band should be considered as a possible cause of a SBO not only in pediatric patients but also in adult patients, even those with no history of abdominal surgery.

## Abbreviations

BR: Band release
C: Childhood
CT: Computed tomography
I: Infant
L-BR: Laparoscopic band release
N: Neonate
SBO: Small bowel obstruction
SR: Segmental resection
US: Ultrasonography
